# Adverse childhood experiences, child poverty, and adiposity trajectories from childhood to adolescence: evidence from the Millennium Cohort Study

**DOI:** 10.1038/s41366-022-01185-1

**Published:** 2022-07-15

**Authors:** Keyao Deng, Rebecca E. Lacey

**Affiliations:** Research Department of Epidemiology and Public Health, University College London, London, UK

**Keywords:** Risk factors, Epidemiology

## Abstract

**Objective:**

This study investigated associations between adverse childhood experiences (ACEs) in early childhood (at ages 9 months and 3 years) and adiposity trajectories of children/adolescents from age 5 to age 17, and the potential interaction between ACEs and poverty on adiposity trajectories.

**Methods:**

Data from the UK Millennium Cohort Study was used. Eight commonly studied ACEs and poverty were measured when the child was aged 9 months and 3 years. ACEs were considered as a cumulative score and as individual experiences. Linear-mixed effect models were employed, modelling BMI and fat mass index (FMI) trajectories from age 5 to 17 (main outcome), adjusting for covariates and stratified by sex. Interactions with poverty were also tested. The sample sizes were 7282 and 6912 for BMI and FMI sample respectively.

**Results:**

Cumulative ACE score was associated with steeper increase in BMI and FMI among boys with 3+ ACEs (BMI: *β* = 0.13, 95% CI: 0.02–0.24; FMI: *β* = 0.09, 95% CI: 0.01–0.19). For individual ACEs, parental depression was associated with steeper increase in BMI/FMI trajectories in both sexes (BMI: boys: *β* = 0.15, 95% CI: 0.07–0.23, girls: *β* = 0.13, 95% CI: 0.05–0.20; FMI: boys: *β* = 0.09, 95% CI: 0.03–0.15, girls: *β* = 0.09, 95% CI: 0.02–0.16). In addition, parental separation and physical punishment were associated with steeper increase in BMI/FMI trajectories among girls (BMI: parental separation: *β* = 0.25; 95% CI: 0.06–0.44, physical punishment: *β* = 0.14; 95% CI: 0.03–0.26; FMI: parental separation: *β* = 0.20; 95% CI: 0.03–0.37, physical punishment: *β* = 0.12; 95% CI: 0.02–0.22). No interaction effect had been found between ACEs and poverty on the adiposity trajectories.

**Conclusions:**

A complex relationship between ACEs in early childhood and adiposity trajectories for children/adolescents was found, highlighting the different effects of specific ACEs and sex differences in the association.

## Introduction

The prevalence of adiposity among children and adolescents has increased over recent decades. In the UK, an estimated 9.7% of children when entering primary school and 20.2% of children in their final year of primary school were classified as obese in 2019 [1]. Adiposity onset during childhood tends to result in both short-term and longitudinal detrimental complications [2]. Several biological, psychosocial, and cultural factors likely contribute to childhood and adolescent adiposity [3], implying that the underlying mechanisms are difficult to disentangle.

Adverse childhood experiences (ACEs) are negative experiences in childhood, including abuse, that require significant adaptation of the developing child [4, 5]. ACEs are highly prevalent; in the UK, almost half of participants in a nationally representative retrospective survey had experienced at least one ACE as a child [6]. A high ACE prevalence was also found cross-nationally, with 39% of participants reporting at least one ACE in an international study involving 21 countries [7]. ACEs are typically considered in research as a cumulative risk score (“ACE score”) which represents the number of adversities reported. Some studies also consider ACEs individually. Both of these approaches have their strengths and limitations [8]. The recommendation from recent research is to move beyond establishing associations between ACE scores and health to explore which specific adversities might be driving associations [9].

Previous research finds long-term detrimental effects of ACEs on physical and mental health outcomes [5, 10]. More specifically, a well-established association between ACEs and adiposity among adult populations was found; meta-analyses suggested that adults who reported ACEs have higher risks of developing obesity during their life-course, with pooled odds ratio ranging from 1.34 to 1.46 [11–13]. A positive dose-response was observed, with an increasing gradient of odds ratio observed as the number of ACEs increases [13]. Changes in health behaviours and the chronic stress response among those experiencing ACEs was suggested as potential explanations for the association [11, 13].

Fewer studies have explicitly focused on ACEs and adiposity within children/adolescents [11, 14–20]. A meta-analysis estimated a pooled odds ratio of 1.13 (95% CI: 0.92–1.39) of the association between child maltreatment and obesity in studies of children/adolescents [11]. In a more recent systematic review, 21 out of 24 studies investigated found an association between ACEs and childhood obesity [14]. However, findings were inconsistent, with some studies finding association only for certain ACEs, just in males or females, in specific age-groups or using certain measures of adiposity. The suggestion from this review is to use multiple methods to verifying ACE exposures when conducting research on ACEs and adiposity. Sex differences in the associations between ACEs and adiposity should also be noticed. Some studies have only found significant associations among girls while other only in boys [15–17], and the underlying mechanisms driving the associations also tend to differ by sex. For example, maternal depression was found to be directly related to elevated BMI among boys but affect girls’ BMI indirectly, mediated via child depression [18]. However, most of the previous studies have used adiposity outcome at a single time point and BMI solely as adiposity measurement, implying that trends in adiposity during childhood/adolescence was uncaptured and potential measurement bias may exist using BMI solely.

Finally, there has been little focus on the role of childhood poverty in investigating the association between ACEs and obesity. Childhood poverty tends to associate with worse health outcomes [21]. Poverty also associates with increasing risks of ACEs [22, 23]. These factors suggest that childhood poverty may interact with ACEs to influence adiposity trajectories, with poverty strengthening the adverse effect of ACEs on adiposity. Moreover, existing literature found that poverty and ACEs overlap [24], suggesting that the effect of these factors cannot be fully understood if analysed independently.

Thus, this study intends to investigate the association between ACEs (both cumulatively and individually) and adiposity trajectories of children from age 5 to 17 years, and the potential interaction effect between ACEs and poverty on adiposity, using both BMI and fat mass index (FMI). We hypothesise that an association exists between higher ACE score and steeper adiposity trajectories, and the strength of association differs with different individual ACEs. We further suggest that an interaction effect exist between ACEs and poverty.

## Methods

### Participants

We used data from the Millennium Cohort Study (MCS), a representative UK cohort of children born between September 2000 and August 2001 [25]. This involved 18,540 children at baseline, with follow up sweeps (MCS2 to MCS7) conducted when the children were aged 3, 5, 7, 11, 14 and 17 years [26]. The percentage of loss of follow up for MCS2 to MCS7 was 15.6%, 17.8%, 25.2%, 28.3%, 36.7% and 42.7% compared to baseline [26]. Non-response was higher for families in ethnic or disadvantaged areas compared with families in advantaged areas [27, 28]. The MCS has ethical approval for all sweeps from the NHS Research Ethics Committee system and obtained informed consent from all participants [29].

### Measures

ACEs: We included eight commonly studied ACEs in early childhood (prior to age 3), as infancy and early childhood were seen as significant period for development [30]. Parental separation: Information on parental separation was derived from the presence of natural mother and natural father in the household in MCS1 (when cohort members/offspring aged 9 months) and MCS2 (when offspring was aged 3). Offspring with parents separated in either wave were classified as yes for parental separation. Parental depression: In MCS1 and MCS2, data on whether parents had ever been diagnosed with depression or serious anxiety was collected from both parents. Offspring with either parent been diagnosed with depression/serious anxiety at either wave was classified as a yes for parental depression. Parental drug use: Data of both parents regarding the frequency of recreational drug use was collected in MCS2. The possible categories were “Occasionally”, “Regularly”, and “Never”. We classified “Occasionally” and “Regularly” as yes and “Never” as no. Offspring with either parent with an affirmative response of drug use was considered as yes for parental drug use. Parental alcohol misuse: Data of both parents regarding the frequency of alcohol consumption was collected in MCS1 and MCS2. The possible categories were “Everyday”, “5–6 times per week”, “3–4 times per week”, “1–2 times per week”, “1–2 times per month”, “Less than once per month” and “Never”. We classified “Everyday” and “5–6 times per week” as yes and all other categories as no. Offspring with either parent having a yes was considered as positive for parental alcohol misuse. Interparental use of force: Data on whether the partner has ever used force in the relationship was collected in MCS1 and MCS2. Offspring with either parent reporting a yes was considered as positive for interparental use of force. Parental discord: Parental discord was asked to both parents in MCS2. Six items were available in the MCS to measure parental discord based on the Golombok Rust Inventory of Marital State (GRIMS) [31]: “Partner is sensitive to and aware of respondents needs”, “Partner does not listen”, “Respondent sometimes feels lonely even with partner”, “Respondent likely to separate from partner”, “How often disagrees over issues concerning child”, and “How happy are you with relationship with partner (a scale with a total score of 7, 1 being most unhappy)”. For the first four items, an affirmative answer was coded as 1 while a non-affirmative was coded as 0. For the item “Respondent sometimes feels lonely even with partner”, a response above several times a week was coded as 1 and for the item “How happy are you with relationship with partner”, a response of 1–3 was coded as 1. We derived a total score of parental discord based on the six items, with a total score >3 considered as yes for parental discord. Harsh parenting: 5 items measuring child maltreatment from the parent-to-child Conflict Tactics Scales (CTSPC) were available [32]. Mothers were asked “How often ignores child if being naughty”, “How often shouts at child when naughty”, “How often send child to bedroom/naughty chair”, “How often take away treats if child being naughty”, “How often tells child off when naughty”, and “How often bribes child when naughty”. For each item, a response of daily, once a week, and once a month was coded as 1 and response of rarely and never was coded as 0. We calculated a total score summing up the 5 items with a total score ≥4 being coded as yes for harsh parenting. Physical punishment: Mothers were asked “How often smacks child when naughty” in MCS2. We classified “Daily”, “Once a week or more”, and “Once a month” as yes and “Rarely” and, “Never” as no. The measurement of parental discord, harsh parenting and physical punishment were consulted from a previous study of ACEs [33]. Finally, we combined all derived ACE measures into a cumulative ACE score with categories 0 ACEs, 1 ACE, 2 ACEs and 3+ ACEs. The distribution of each of the ACE and ACE score was summarised in Table 1.

**Table 1:** Descriptive statistics of the Millennium Cohort Study (MCS) sample.

	Total sample, MCS 1^a^ *N* = 18,540		BMI sample^b^ *N* = 7282		FMI sample^c^ *N* = 6912	
	Boys, *N* = 9527 N/mean; %/SD	Girls, *N* = 9013 N/mean; %/SD	Boys, *N* = 3711 N/mean; %/SD	Girls, *N* = 3571 N/mean; %/SD	Boys, *N* = 3514 N/mean; %/SD	Girls, *N* = 3398 N/mean; %/SD
*Adverse childhood experiences (ACEs)*						
Parental separation; No	5854; 61.45	5674; 62.95	3530; 95.12	3417; 95.69	3344; 95.16	3256; 95.82
Yes	1730; 18.16	1624; 18.02	181; 4.88	154; 4.31	170; 4.84	142; 4.18
Missing	1943; 20.39	1715; 19.03	–	–	–	–
Parental depression; No	6083; 63.85	5703; 63.28	2428; 65.43	2306; 64.58	2295; 65.31	2189; 64.42
Yes	3444; 36.15	3309; 36.71	1283; 34.57	1265; 35.42	1219; 34.69	1209; 35.58
Missing	0; 0.00	1; 0.01	–	–	–	–
Parental drug use; No	6432; 67.51	6210; 68.90	3391; 91.38	3269; 91.54	3218; 91.58	3105; 91.38
Yes	606; 6.36	562; 6.24	320; 8.62	301; 8.46	296; 8.42	293; 8.62
Missing	2489; 26.13	2241; 24.86	–	–	–	–
Parental alcohol misuse; No	8095; 84.97	7635; 84.71	2875; 77.47	2778; 77.79	2716; 77.29	2638; 77.63
Yes	1432; 15.03	1377; 15.28	836; 22.53	793; 22.21	798; 22.71	760; 22.37
Missing	0; 0.00	1; 0.01	–	–	–	–
Interparental use of force; No	6830; 71.69	6459; 71.66	3138; 84.56	3013; 84.37	2977; 84.72	2859; 84.14
Yes	1094; 11.48	1053; 11.68	573; 15.44	558; 15.63	537; 15.28	539; 15.86
Missing	1 603; 16.83	1 501; 16.65	–	–	–	–
Parental discord; No	5401; 56.69	5195; 57.64	3407; 91.81	3330; 93.25	3223; 91.72	3162; 93.05
Yes	475; 4.99	433; 4.80	304; 8.19	241; 6.75	291; 8.28	236; 6.95
Missing	3651; 38.32	3385; 37.56	–	–	–	–
Harsh parenting; No	4310; 45.24	4396; 48.77	2584; 69.63	2657; 74.40	2436; 69.32	2524; 74.28
Yes	1746; 18.33	1 386; 15.38	1 127; 30.37	914; 25.60	1 078; 30.68	874; 25.72
Missing	3471; 36.43	3 231; 35.85	–	–	–	–
Physical punishment; No	5493; 57.66	5559; 61.68	3049; 82.16	3150; 88.21	2884; 82.07	2987; 87.90
Yes	1090; 11.44	752; 8.34	662; 17.84	421; 11.79	630; 17.93	411; 12.10
Missing	2944; 30.90	2702; 29.98	–	–	–	–
*ACE score*						
0	1226; 12.87	1263; 14.01	915; 24.66	974; 27.28	859; 24.45	918; 27.02
1	1668; 17.51	1677; 18.61	1242; 33.47	1276; 35.73	1180; 33.58	1204; 35.43
2	1180; 12.39	1077; 11.95	904; 24.36	814; 22.79	857; 24.39	786; 23.13
3+	899; 9.44	711; 7.89	650; 17.52	507; 14.20	618; 17.59	490; 14.42
Missing	4 554; 47.80	4 285; 47.54	–	–	–	–
Poverty; No	5571; 58.48	5313; 58.95	3087; 83.19	2996; 83.90	2930; 83.38	2859; 84.14
Yes	3529; 37.04	3318; 36.81	624; 16.81	575; 16.10	584; 16.62	939; 15.86
Missing	427; 4.48	382; 4.24	–	–	–	
*BMI (age)*						
5 years	16.40; 1.92	16.32; 1.88	16.42; 1.84	16.27; 1.76	–	–
7 years	16.61; 2.33	16.70; 2.39	16.57; 2.16	16.61; 2.23	–	–
11 years	19.06; 3.59	19.46; 3.75	18.92; 3.38	19.23; 3.50	–	–
14 years	20.97; 4.05	21.96; 4.17	20.79; 3.81	21.69; 3.90	–	–
17 years	23.01; 4.62	23.57; 4.83	22.80; 4.31	23.37; 4.60	–	–
*FMI (age)*						
7 years	3.45; 1.48	3.83; 1.59	–	–	3.38; 1.36	3.74; 1.46
11 years	4.09; 2.49	5.05; 2.52	–	–	3.94; 2.25	4.87; 2.35
14 years	3.83; 2.73	6.22; 2.88	–	–	3.62; 2.46	5.99; 2.71
17 years	4.09; 3.04	6.97; 3.52	–	–	3.85; 2.75	6.81; 3.34
*Ethnicity*						
White	7876; 82.67	7401; 82.11	3457; 93.16	3290; 92.13	3274; 93.17	3131; 92.14
Mixed/Other	406; 4.26	413; 4.58	83; 2.24	108; 3.02	78; 2.22	102; 3.00
Indian	241; 2.53	224; 2.49	62; 1.67	59; 1.65	59; 1.68	56; 1.65
Pakistani and Bangladeshi	632; 6.63	633; 7.02	65; 1.75	80; 2.24	63; 1.79	78; 2.30
Black or Black British	348; 3.65	318; 3.53	44; 1.19	34; 0.95	40; 1.14	31; 0.91
Missing	24; 0.25	24; 0.27	–	–	–	–
Birth weight	3.40; 0.60	3.29; 0.57	3.50; 0.56	3.36; 0.56	3.50; 0.56	3.36; 0.56
Maternal prenatal BMI	23.66; 4.48	23.63; 4.44	23.90; 4.30	23.86; 4.23	23.91; 4.29	23.89; 4.25
*Parental social class*						
Semi routine and routine	1542; 16.19	1384; 15.36	588; 15.84	525; 14.70	540; 15.37	494; 14.54
Lower supervisory and technical	764; 8.02	713; 7.91	374; 10.08	358; 10.03	350; 9.96	339; 9.98
Small employers	638; 6.70	595; 6.60	296; 7.98	291; 8.15	279; 7.94	275; 8.09
Intermediate	822; 8.63	838; 9.30	407; 10.97	450; 12.60	386; 10.98	428; 12.60
Managerial/professional	3283; 34.46	3104; 34.44	2046; 55.13	1947; 54.52	1959; 55.75	1826; 54.80
Missing	2478; 26.01	2379; 26.40	–	–	–	
*Parental education*						
None	1318; 13.83	1260; 13.98	118; 3.18	113; 3.16	104; 2.96	106; 3.12
GCSEs	4051; 42.52	3688; 40.92	1385; 37.32	1321; 36.99	1316; 37.45	1249; 36.76
A Levels	905; 9.50	884; 9.81	407; 10.97	419; 11.73	385; 10.96	399; 11.74
Higher education	3042; 31.93	2957; 32.81	1779; 47.94	1696; 47.49	1691; 48.12	1624; 47.79
Oversea qualifications	190; 1.99	205; 2.27	22; 0.59	22; 0.62	18; 0.51	20; 0.59
Missing	21; 0.22	19; 0.21	–	–	–	

^a^Sample of Millennium (MCS) cohort members at wave 1 (9 months).

^b^Sample of MCS cohort members at wave, with complete data for adverse childhood experiences, all covariates and at least one BMI measure.

^c^Sample of MCS cohort members at wave, with complete data for adverse childhood experiences, all covariates and at least one fay mass index (FMI) measure.

Poverty: The McClements below 60% median poverty indicator was used (MCS1 and MCS2) and those below the 60% median were classified yes for poverty. We used mainly MCS1 data and supplemented data from MCS2 if the former was missing, which involved 334 (4.58%) and 317 (4.59%) participants of the BMI and FMI sample respectively.

Adiposity: We used Body Mass Index (BMI) and FMI as measures of adiposity. Height (to the nearest 0.1 cm) and weight measurements (to the nearest 0.1 kg) were taken by trained interviewers when cohort members were aged 5, 7, 11, 14 and 17 (MCS3 to MCS7). BMI was then calculated by weight over the square of height (kg/m^2^). Body fat percentage (BFP, measured to the nearest 0.1%) were measured when cohort members were aged 7, 11, 14 and 17 (MCS4 to MCS7). Height, weight and BFP was measured by Tanita scales (BF-522W), with BFP been measured by sending a weak electrical current around the body from one foot to the other [26]. The Tanita scales have been confirmed to have high validity and reliability, with the scale having ±5% accuracy compared to the institutional standard of body composition analysis and having ±1% variation for repeated measures [34]. We then calculated the FMI using the equation FMI = BF/height^2^.

Covariates: Offspring’s sex, ethnicity (White; Mixed and Other; Indian; Pakistani and Bangladeshi; Black/Black British) and birth weight (in kilograms) were included as cohort member-related covariates. Mother’s self-reported prenatal BMI (kg/m^2^) and age at birth of offspring recorded in MCS1 were included. Parental occupational social class as measured by the National Statistics Socioeconomic Classification five category version (Semi routine and routine; Lower supervisory and technical; Small employers; Intermediate; Managerial and professional) and parental highest qualification (None; GCSEs; A-Levels; Higher education; Overseas qualifications) were also included. For parental social class and parental highest education, the highest category among the parents was used for two-parent households. All covariates listed above were included in the models.

### Statistical analyses

**Fig. 1:**
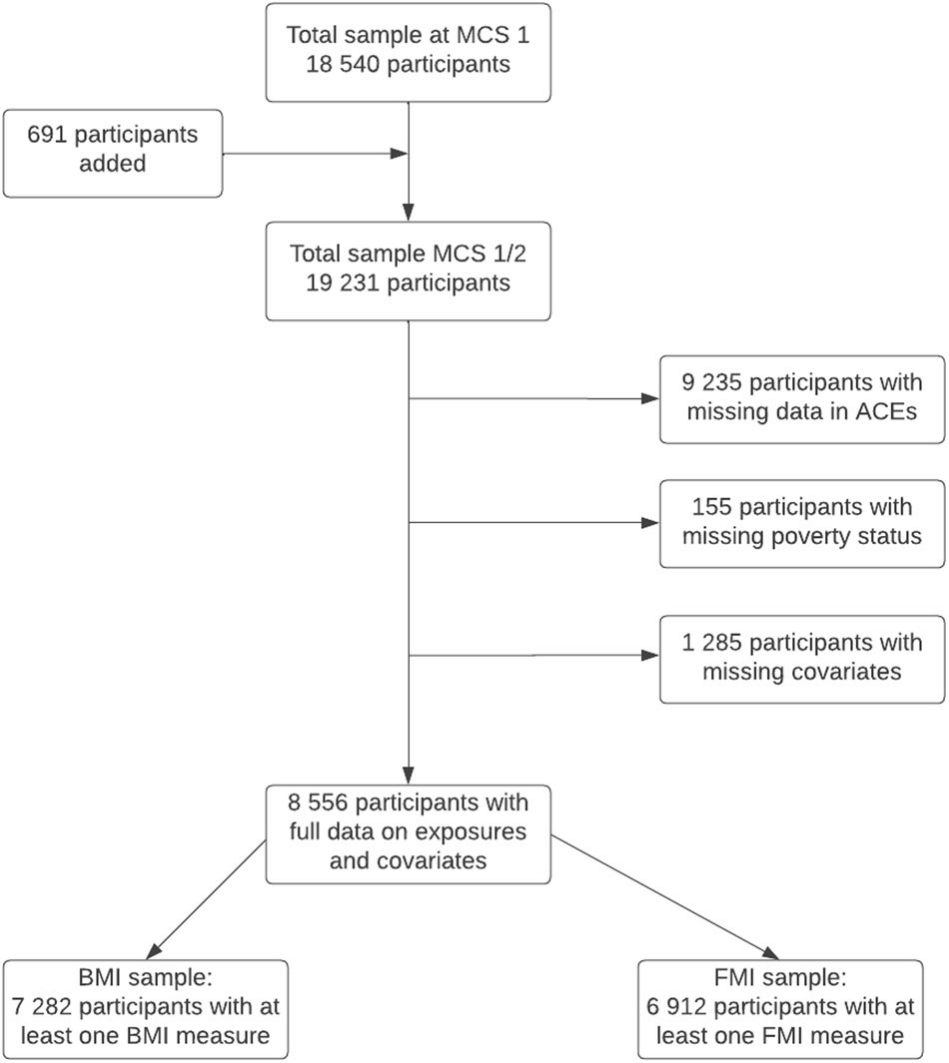
Analytical sample selection process. A flow chart summarising the derivation of the study sample of the Millennium Cohort Study.

Linear-mixed effects models were used to analyse the association between ACE score/each individual ACE, and each of the outcomes of BMI and FMI. In the case of BMI, the intercept was set at wave 3 (age 5) and trajectories modelled through age 17. For FMI the intercept was set at wave 4 (age 7) and trajectories modelled through age 17. The best fitting models were those with random slopes and intercepts for both outcomes. In addition, a quadratic term for time improved model fit. Model fit was assessed by comparing Akaike information criterion and Bayesian information criterion terms. Maximum likelihood estimation and complete-case analysis was applied, meaning that offspring with at least one adiposity measurement and complete data in ACEs, poverty, and covariates were included in the analysis. This resulted in a sample size of 7282 children for the BMI trajectory analysis and 6912 children for the FMI trajectory analysis (see diagram Fig. 1). Interaction terms between ACE scores/individual ACEs and poverty and sex were tested using a three-way interaction term of ACEs, time and poverty or an interaction term for time and sex. We ran all analyses using STATA 17 (StataCorp LLC) [35].

## Results

### Descriptive characteristics

About 25%, 33%, 24% and 18% boys and 27%, 36%, 23% and 14% girls reported 0, 1, 2, 3+ ACEs respectively (Table 1). The most common ACE was parental depression for both sexes/samples. Boys were more likely to experience harsh parenting and physical punishment but no other differences in ACEs by sex were observed. About 16% of the sample was living in households with poverty. Both BMI and FMI increased with age. The mean BMI was 16.42 (SD = 1.84) for boys and 16.27 (SD = 1.76) for girls at age 5 and reached 22.80 (SD = 4.31) for boys and 23.37 (SD = 4.60) at age 17. For FMI, the mean value for boys was 3.38 (SD = 1.36) at age 7 and rise to 3.85 (SD = 2.75) at age 17. The rise in FMI was steeper among girls, with mean FMI of 3.74 (SD = 1.46) at age 7–6.81 (SD = 3.34) at age 17. Most children were of white ethnicity, with parents of managerial and professional occupations and with higher education qualifications. Supplementary table 1 summarises ACEs and BMI/FMI measures according to poverty status. Those with poverty were more likely to have 3+ ACEs and have higher BMI/FMI from age 11.

### Associations between ACE score and BMI/FMI trajectories

Table 2 presents the results of mixed-effects models of the association between ACE score and BMI/FMI trajectories, accounting for all covariates. The association between ACE score and BMI/FMI trajectories are 0.03 and 0.02 smaller among girls. As associations were found to differ by sex, all models were stratified by sex. Overall, there was a positive association between ACE scores and steeper BMI/FMI trajectories. However, this association was only significant for boys with 3+ ACEs. Boys with 3+ ACEs had a steeper increase in both BMI (*β* = 0.13, 95% CI: 0.02–0.24) and FMI (*β* = 0.09, 95% CI: 0.01–0.19) compared to boys with 0 ACEs. No differences in the intercept for BMI (age 5) and FMI (age 7) were observed by ACE score.

**Table 2:** Adjusted longitudinal mixed effect model of the association between ACE score and BMI/FMI trajectories.

	BMI trajectories (5–17 years)				FMI trajectories (7–17 years)			
	Boys		Girls		Boys		Girls	
	*β*	95% CI	*β*	95% CI	*β*	95% CI	*β*	95% CI
*Baseline*								
0 ACEs (ref)								
1 ACE	−0.06	−0.22–0.09	0.02	−0.13–0.17	−0.01	−0.14–0.12	0.01	−0.12–0.15
2 ACEs	0.05	−0.12–0.22	−0.10	−0.26–0.08	0.07	−0.07–0.22	−0.08	−0.23–0.07
3+ ACEs	0.13	−0.05–0.32	0.03	−0.16–0.23	0.07	−0.09–0.23	0.04	−0.13–0.21
Intercept (5/7 years)	16.41	16.04–16.78	16.30	15.92–16.67	3.91	3.58–4.24	3.98	3.64–4.32
*Rate of change*								
0 ACEs (ref)								
1 ACE	0.07	−0.03–0.17	0.07	−0.02–0.16	0.05	−0.03–0.13	0.07	−0.01–0.16
2 ACEs	0.06	−0.04–0.17	0.02	−0.08–0.12	0.01	−0.07–0.09	0.04	−0.05–0.13
3+ ACEs	0.13*	0.02–0.24	0.06	−0.06–0.18	0.09*	0.01–0.19	0.02	−0.09–0.12
Time/Slope	0.60	0.51–0.69	1.07	0.98–1.16	0.37	0.28–0.45	1.31	1.22–1.39
Time squared	0.27	0.26–0.29	0.23	0.21–0.24	−0.08	−0.10–−0.06	−0.08	−0.10–−0.06
*Variance*								
Variance: slope	0.85	0.79–0.90	0.82	0.76–0.88	0.37	0.33–0.41	0.58	0.54–0.63
Variance: intercept	1.74	1.59–1.90	1.70	1.55–1.86	1.35	1.23–1.47	1.66	1.54–1.79
Covariance	0.42	0.35–0.49	0.60	0.53–0.67	0.46	0.41–0.51	0.54	0.48–0.59

*P < 0.05, using mixed-effects models.

### Associations between individual ACEs and BMI/FMI trajectories

Table 3 presents the results of adjusted mixed-effect models of the associations between individual ACEs and BMI and FMI trajectories. For both BMI and FMI, we observed a steeper increase for boys and girls who reported parental depression (BMI: boys: *β* = 0.15, 95% CI: 0.07–0.23, girls: *β* = 0.13, 95% CI: 0.05–0.20; FMI: boys: *β* = 0.09, 95% CI: 0.03–0.15, girls: *β* = 0.09, 95% CI: 0.02–0.16). In addition, boys with parental depression had higher starting values of BMI and FMI. On the contrary, we observed a decline in slope of BMI and FMI trajectories for both sexes with parental alcohol misuse (BMI: boys: *β* = −0.11; 95% CI: −0.20–−0.02, girls: *β* = −0.18, 95% CI: −0.27–−0.09; FMI: boys: *β* = −0.07; 95% CI: −0.14–−0.01, girls: *β* = −0.16, 95% CI: −0.23–−0.08). We also observed steeper slope in BMI and FMI for girls who experienced parental separation and physical punishment (BMI: parental separation: *β* = 0.25; 95% CI: 0.06–0.44, physical punishment: *β* = 0.14; 95% CI: 0.03–0.26; FMI: parental separation: *β* = 0.20; 95% CI: 0.03–0.37, physical punishment: *β* = 0.12; 95% CI: 0.02–0.22), and boys who experienced parental separation additionally started with higher FMI values at age 7. Finally, the experience of interparental use of force was associated with higher intercept values of BMI and FMI for girls and FMI for boys.

**Table 3:** Adjusted longitudinal mixed effect model of the association between individual ACEs and BMI/FMI trajectories.

	BMI trajectories (5–17 years)				FMI trajectories (7–17 years)			
Individual ACEs (ref: no)	Boys		Girls		Boys		Girls	
	*β*	95% CI	*β*	95% CI	*β*	95% CI	*β*	95% CI
Parental separation	0.10	−0.19–0.38	−0.03	−0.34–0.27	0.25*	0.01–0.50	−0.02	−0.30–0.26
Parental separation*time	0.14	−0.03–0.32	0.25*	0.06–0.44	0.05	−0.10–0.19	0.20*	0.03–0.37
Time/slope	0.66	0.59–0.72	1.10	1.03–1.17	0.40	0.34–0.47	1.36	1.27–1.40
Time squared	0.27	0.26–0.29	0.23	0.21–0.24	−0.08	−0.10–−0.06	−0.08	−0.10–−0.06
Intercept	16.42	16.06–16.78	16.30	15.94–16.66	3.90	3.58–4.22	3.98	3.54–4.31
Parental depression	0.12*	0.01–0.25	−0.03	−0.15–0.10	0.15*	0.04–0.26	0.31	−0.08–0.14
Parental depression*time	0.15*	0.07–0.23	0.13*	0.05–0.20	0.09*	0.03–0.15	0.09*	0.02–0.16
Time/slope	0.61	0.54–0.68	1.07	0.99–1.14	0.37	0.30–0.44	1.31	1.24–1.38
Time squared	0.27	0.26–0.29	0.23	0.21–0.24	−0.08	−0.10–−0.06	−0.08	−0.10–−0.06
Intercept	16.37	16.02–16.73	16.31	15.95–16.68	3.87	3.55–4.19	3.96	3.63–4.30
Parental drug use	0.10	−0.10–0.31	−0.01	−0.20–0.21	0.01	−0.18–0.19	0.02	−0.17–0.21
Parental drug use*time	0.01	−0.12–0.14	−0.04	−0.18–0.09	0.02	−0.08–0.13	−0.07	−0.19–0.05
Time/slope	0.66	0.59–0.73	1.12	1.05–1.18	0.40	0.34–0.47	1.35	1.28–1.42
Time squared	0.27	0.26–0.29	0.22	0.21–0.24	−0.08	−0.10–−0.06	−0.08	−0.10–−0.06
Intercept	16.43	16.07–16.78	16.30	15.94–16.65	3.95	3.64–4.27	3.97	3.65–4.30
Parental alcohol misuse	0.01	−0.13–0.15	−0.03	−0.18–0.11	−0.06	−0.18–0.06	−0.10	−0.23–0.03
Parental alcohol misuse*time	−0.11*	−0.20–−0.02	−0.18*	−0.27–−0.09	−0.07*	−0.14–−0.01	−0.16*	−0.23–−0.08
Time/slope	0.69	0.62–0.76	1.15	1.08–1.22	0.42	0.35–0.49	1.38	1.31–1.45
Time squared	0.27	0.26–0.29	0.22	0.21–0.24	−0.08	−0.10–−0.06	−0.08	−0.10–−0.06
Intercept	16.44	16.09–16.79	16.30	15.94–16.66	3.96	3.65–4.28	3.99	3.66–4.32
Interparental use of force	0.14	−0.02–0.30	0.22*	0.06–0.38	0.15*	0.01–0.29	0.18*	0.04–0.33
Interparental use of force*time	0.07	−0.03–0.17	−0.01	−0.10–0.10	0.03	−0.05–0.11	−0.05	−0.14–0.04
Time/slope	0.65	0.58–0.72	1.11	1.04–1.18	0.40	0.33–0.47	1.35	1.28–1.42
Time squared	0.27	0.26–0.29	0.23	0.21–0.24	−0.08	−0.10–−0.06	−0.08	−0.10–−0.06
Intercept	16.42	16.06–16.77	16.27	15.91–16.62	3.93	3.61–4.24	3.95	3.62–4.28
Parental discord	0.08	−0.13–0.30	0.08	−0.15–0.32	0.06	−0.12–0.24	−0.04	−0.25–0.17
Parental discord*time	0.07	−0.06–0.20	−0.07	−0.22–0.07	0.08	−0.02–0.20	−0.04	−0.17–0.09
Time/slope	0.66	0.59–0.73	1.12	1.05–1.19	0.40	0.33–0.46	1.35	1.28–1.41
Time squared	0.27	0.26–0.29	0.22	0.21–0.24	−0.08	−0.10–−0.06	−0.08	−0.10–−0.06
Intercept	16.44	16.08–16.79	16.29	15.93–16.64	3.95	3.63–4.27	3.98	3.65–4.31
Harsh parenting	−0.11	−0.23–0.02	−0.09	−0.23–0.04	−0.07	−0.18–0.04	−0.07	−0.19–0.05
Harsh parenting*time	0.06	−0.02–0.14	−0.01	−0.09–0.08	0.03	−0.03–0.10	0.01	−0.06–0.09
Time/slope	0.64	0.57–0.72	1.11	1.04–1.18	0.39	0.32–0.46	1.34	1.27–1.41
Time squared	0.27	0.26–0.29	0.22	0.21–0.24	−0.08	−0.10–−0.06	−0.08	−0.10–−0.06
Intercept	16.47	16.12–16.83	16.31	15.95–16.66	3.97	3.66–4.29	3.99	3.66–4.31
Physical punishment	0.11	−0.05–0.26	−0.04	−0.22–0.14	−0.03	−0.15–0.11	−0.01	−0.17–0.16
Physical punishment*time	0.02	−0.08–0.11	0.14*	0.03–0.26	0.03	−0.05–0.11	0.12*	0.02–0.22
Time/slope	0.66	0.59–0.73	1.09	1.03–1.16	0.40	0.33–0.47	1.33	1.26–1.39
Time squared	0.27	0.26–0.29	0.23	0.21–0.24	−0.08	−0.10–−0.06	−0.08	−0.10–−0.06
Intercept	16.42	16.06–16.77	16.30	15.94–16.66	3.96	3.64–4.28	3.98	3.65–4.30

*P < 0.05, using mixed-effects models.

### Poverty as an effect modifier between ACE and BMI/FMI trajectories

Supplementary tables 4, 5 presents the adjusted models of ACE score/individual ACEs and BMI/FMI trajectories, with poverty interactions. We found no interactions by poverty. However, children with poverty have steeper increase in both BMI and FMI compared to those without poverty, among the individual ACE models.

## Discussion

In line with other studies, our findings suggested a high prevalence of ACEs in the MCS, with about three-quarters of the children reported having at least one ACE by age 3. For the analyses using the ACE score, a less consistent relationship between ACE score and adiposity was found compared to previous studies. There was no association between ACE score and BMI and FMI at baseline (at age 5/7), but some evidence that there was an association between ACE score and steeper increase in BMI and FMI trajectories from age 5 to 17. However, this association was only significant for boys with 3+ ACEs. This was consistent with several studies which suggested no association between cumulative ACEs and obesity or only found the association among boys [36–38]. However, these studies investigated adiposity at one time point rather than a longitudinal adiposity trajectory of children, which the current study does. The results of no association between ACE score and adiposity trajectories (except for boys with 3+ ACEs) can potentially be explained by the existence of a latent period suggested by some authors, which indicates that the effect of ACEs on children’s adiposity development may require some time to manifest into biological change [14, 39]. A longitudinal study analysing the association between childhood maltreatment and long-term BMI trajectories had further confirmed this, as an association was only observed until the participants reach mid-adulthood [40].

Different results have been found for the associations between individual ACEs and adiposity trajectories. Contrary to our expectation, children who reported parental alcohol misuse had a flatter increase in adiposity trajectories. This can probably be explained by the measurement of parental alcohol misuse. Due to data availability, we used the data on frequency of alcohol consumption as the measurement of alcohol misuse and defined consuming alcohol daily or 5–6 times per week as misuse. Quantity of alcohol consumed was uncaptured. Previous literature has suggested that higher socioeconomic position was associated with more frequent alcohol consumption but lower quantities on each drinking occasion [41], hence the inverse relationship found between parental alcohol use and adiposity trajectories can be related to frequent drinkers having higher socioeconomic positions.

Sex differences of associations between ACE score and adiposity trajectories observed can be explained by several mechanisms. From a sociocultural perspective, differences in seeking social support among girls and boys can potentially explain this. Previous evidence suggested that girls were more proactive in seeking social support from peers and adults compared to boys [42]. Social support may benefit health and potentially reduce the detrimental effect of ACEs. For example, it was suggested that social support from parents have an important influence on adolescents’ physical activity-related behaviours [43], which can affect adolescents’ adiposity trajectories. Moreover, ACEs were associated with elevated risk of restrictive eating disorders [44, 45], which tend to be more common among females [46, 47]. Perhaps no association between ACEs and adiposity trajectories found among girls was due to restriction and compensatory behaviours. Apart from the social aspect, sex differences observed can also be explained using a biological pathway. It was suggested that ACEs can lead to the hyperactivation of the stress system, which reduces the cortisol level hence accelerate the onset of puberty [48]. This attenuation in cortisol was only found in boys experiencing maltreatment.

Sex differences were also found when investigating individual ACEs. While no significant association has been found between ACE score and adiposity trajectories among girls, four individual ACEs were associated with adiposity compared to two individual ACEs in boys. This highlights the differences in the approaches to measuring ACEs. While the cumulative ACE score summarised the adversities a child experienced in multiple domains, it assumes that each adversity is equally important and ignores the potentially different effects of individual ACEs [8]. Perhaps the finding of no associations between ACE score and adiposity trajectories was due to a dilution effect, with the effect of certain ACEs being attenuated due to other ACEs. The two individual ACEs that were found to be associated with a steeper increase in adiposity among girls but not in boys were parental separation and physical punishment. Some studies found that parental divorce/separation affects boys and girls differently, with girls who experienced parental divorce having a higher likelihood of developing anxiety and depression [49, 50]. As there is a well-established reciprocal relationship between depression and adiposity [51], this may explain the steeper increase in adiposity among girls who reported parental separation but not among boys who experienced parental separation. A relationship between experiencing physical punishment and steeper increase in adiposity was also only found in girls. Physical punishment tends to associate with internalising problems such as depression in girls rather with externalising behaviours, which tend to be more associated with obesity and related unhealthy behaviours [50].

Our findings found no interaction between poverty and ACEs on adiposity trajectories. Previous studies suggested that poverty is an important risk factor of ACEs [52], which implies that poverty increase the likelihood of experiencing ACEs but did not modify the detrimental effect of ACEs on health. This is consistent with our findings that associations between ACEs and adiposity exist across both levels of socioeconomic disadvantage. Moreover, we find that poverty solely was associated with greater slope in BMI/ FMI trajectories among individual ACE models but not in ACE score models, suggesting that ACEs were more predictive of adiposity.

The present study has several strengths. First, a large nationally representative sample was employed, indicating that results tend to be generalisable to a large population. Linear mixed-effect models were used for the analysis, which allows adiposity trajectories to be analysed while considering both the intra- and interindividual variation. Moreover, the usage of linear mixed-effect models allows those with at least one observation of BMI/FMI to be included, which ensure a large study sample to be obtained. In respect of the measurements, ACEs were reported prospectively, which reduce the likelihood of recall bias. Finally, the outcome of adiposity was measured by both BMI and FMI, which can distinguish between fat mass and lean mass and ensure that changes in adiposity were accurately measured [53].

However, several limitations of this study should be noted. ACE score and individual ACEs were used as the measurement of ACE. As previously mentioned, these two measurements have their own strengths and weaknesses. The assumption that each ACE is equally important for outcomes made by the ACE score approach and the ignorance of co-occurrence of ACEs by using single ACE as measurement can be potentially problematic [8]. Future studies can employ person-centred approaches such as the latent class analysis model to investigate the impact of different ACE patterns, however previous research using latent class analysis found that this did not work in the MCS [28]. Apart from the operationalisation of ACE approaches, some other measurement bias of ACEs exists. As discussed above, the measurement of parental alcohol misuse needs to be treated as an under-estimation. Moreover, only some of the items were available for measuring parental discord and harsh parenting instead of the original GRIMS and Stratus scale. As ACEs was reported by parents, reporting bias may exist especially for item such as parental drug use and adversities outside the family such as bullying was uncaptured. These can result in misclassification and underestimate of ACEs. Apart from measurement bias, a complete-case analysis was used, implying that our study sample was restricted to those with complete information in ACEs, poverty, covariates, and at least one adiposity observation, which may lead to potential attrition bias. Finally, this study has only investigated associations, hence the results cannot derive any causal inferences.

## Conclusion

Overall, our studies found some evidence on associations between ACEs and adiposity trajectories among children/adolescents. Using ACE score, ACEs were associated with steeper increase in both BMI and FMI trajectories among boys with three or more ACEs. Using individual ACEs, parental depression for both sexes and parental separation and physical punishment in girls were found to be associated with steeper increase in adiposity, while parental alcohol misuse was associated with flatter increase in adiposity in both sexes. Parental depression in boys and interparental use of force in both sexes were found to had higher intercept of BMI/FMI. No interaction effect had been found between ACEs and poverty on the adiposity trajectories. Together, these findings have shown a complicated relationship between ACEs and adiposity trajectories and highlighted the sex differences and using different operationalisation of ACEs. Future studies might investigate the potential mechanisms explaining the complex relationships found between ACEs and adiposity among children/adolescents.

## Supplementary information

Supplemental materials

STROBE checklist

## Data Availability

The datasets analysed in this study are available from the UK Data Service website: beta.ukdataservice.ac.uk/datacatalogue/series/series?id=2000031.
