# Supplementary material for: Adverse childhood experiences, child poverty, and adiposity trajectories from childhood to adolescence: evidence from the Millennium Cohort Study
Source: Int J Obes (Lond). 2022 Jul 15;46(10):1792–800. doi: 10.1038/s41366-022-01185-1 (PMC9492536; doi:10.1038/s41366-022-01185-1)

Supplementary table 1. Comparison of ACEs and adiposity according to poverty status for BMI and FMI sample

|  | BMI sample  N=7 282 | | FMI sample  N=6 912 | |
| --- | --- | --- | --- | --- |
|  | Poverty (No),  N=6 083 (83.53%)  N/mean; %/SD | Poverty (Yes),  N=1 199 (16.47%)  N/mean; %/SD | Poverty (No),  N=5 789 (83.75%)  N/mean; %/SD | Poverty (Yes),  N=1 123 (16.25%)  N/mean; %/SD |
| Adverse childhood experiences (ACEs) | |  |  |  |
| Parental separation; No | 5 947; 97.76 | 1 000; 83.40* | 5 661; 97.79 | 939; 83.62* |
| Yes | 136; 2.24 | 199; 16.60 | 128; 2.21 | 184; 16.38 |
| Parental depression; No | 4 067; 66.86 | 667; 55.63* | 3 861; 66.70 | 623; 55.48* |
| Yes | 2 016; 33.14 | 532; 44.37 | 1 928; 33.30 | 500; 44.52 |
| Parental drug use; No | 5 608; 92.19 | 1 052; 87.74* | 5 338; 92.21 | 985; 87.71* |
| Yes | 475; 7.81 | 147; 12.26 | 451; 7.79 | 138; 12.29 |
| Parental alcohol misuse; No | 4 632; 76.15 | 1 021; 85.15* | 4 397; 75.95 | 957; 85.22* |
| Yes | 1 451; 23.85 | 178; 14.85 | 1 392; 24.05 | 166; 14.78 |
| Interparental use of force; No | 5 158; 84.79 | 993; 82.82 | 4 906; 84.75 | 930; 82.81 |
| Yes | 925; 15.21 | 206; 17.18 | 883; 15.25 | 193; 17.19 |
| Parental discord; No | 5 654; 92.95 | 1 083; 90.33* | 5 373; 92.81 | 1 012; 90.12* |
| Yes | 429; 7.05 | 116; 9.67 | 416; 7.19 | 111; 9.88 |
| Harsh parenting; No | 4 310; 70.85 | 931; 77.65* | 4 091; 70.67 | 869; 77.38* |
| Yes | 1 773; 29.15 | 268; 22.35 | 1 698; 29.33 | 254; 22.62 |
| Physical punishment; No | 5 188; 85.29 | 1 011; 84.32 | 4 924; 85.06 | 947; 84.33 |
| Yes | 895; 14.71 | 188; 15.68 | 865; 14.94 | 176; 15.67 |
|  |  |  |  |  |
| ACE score |  |  |  |  |
| 0 | 1 610; 26.47 | 279; 23.27* | 1 513; 26.14 | 264; 23.51* |
| 1 | 2 136; 35.11 | 382; 31.86 | 2 032; 35.10 | 352; 31.34 |
| 2 | 1 428; 23.48 | 290; 24.19 | 1 368; 23.63 | 275; 24.49 |
| 3+ | 909; 14.94 | 248; 20.68 | 876; 15.13 | 232; 20.66 |
|  |  |  |  |  |
| BMI (age) |  |  |  |  |
| 5 years | 16.35; 1.80 | 16.28; 1.83 | - | - |
| 7 years | 16.58; 2.15 | 16.65; 2.43 | - | - |
| 11 years | 19.02; 3.38 | 19.35; 3.76* | - | - |
| 14 years | 21.16; 3.79 | 21.64; 4.31* | - | - |
| 17 years | 23.00; 4.35 | 23.61; 5.11* | - | - |
| FMI (age) |  |  |  |  |
| 7 years | - | - | 3.55; 1.39 | 3.63; 1.59 |
| 11 years | - | - | 4.35; 2.29 | 4.67; 2.65* |
| 14 years | - | - | 4.73; 2.78 | 5.14; 3.16* |
| 17 years | - | - | 5.26; 3.31 | 5.80; 3.84* |

* P < 0.05, using chi-square, t-test or t-test of trend across poverty status

Supplementary table 2. Unadjusted longitudinal mixed effect model of the association between ACE score and BMI/FMI trajectories

|  | BMI trajectories (5-17 years) | | | | FMI trajectories (7-17 years) | | | |
| --- | --- | --- | --- | --- | --- | --- | --- | --- |
|  | Boys | | Girls | | Boys | | Girls | |
|  | β | 95% CI | β | 95% CI | β | 95% CI | β | 95% CI |
| **Baseline** |  |  |  |  |  |  |  |  |
| 0 ACEs (ref) |  |  |  |  |  |  |  |  |
| 1 ACE | -0.07 | -0.23- 0.09 | -0.01 | -0.16- 0.15 | -0.04 | -0.18- 0.10 | -0.01 | -0.15- 0.13 |
| 2 ACEs | 0.02 | -0.15- 0.19 | -0.10 | -0.27- 0.08 | 0.01 | -0.15- 0.15 | -0.09 | -0.25- 0.06 |
| 3+ ACEs | 0.14 | -0.05- 0.33 | 0.02 | -0.18- 0.22 | 0.05 | -0.11- 0.22 | 0.02 | -0.16- 0.20 |
| Intercept (5/7 years) | 16.21 | 16.09-16.33 | 15.99 | 15.87-16.11 | 3.46 | 3.36- 3.57 | 3.74 | 3.63- 3.85 |
| **Rate of change** |  |  |  |  |  |  |  |  |
| 0 ACEs (ref) |  |  |  |  |  |  |  |  |
| 1 ACE | 0.07 | -0.03- 0.17 | 0.07 | -0.02- 0.16 | 0.05 | -0.02- 0.13 | 0.07 | -0.01- 0.16 |
| 2 ACEs | 0.06 | -0.05- 0.16 | 0.02 | -0.09- 0.12 | 0.01 | -0.07- 0.09 | 0.04 | -0.05- 0.13 |
| 3+ ACEs | 0.13* | 0.01- 0.24 | 0.06 | -0.06- 0.18 | 0.09* | 0.01- 0.19 | 0.02 | -0.09- 0.12 |
| Time/Slope | 0.60 | 0.51- 0.70 | 1.08 | 0.98- 1.16 | 0.37 | 0.28- 0.45 | 1.31 | 1.22- 1.39 |
| Time squared | 0.27 | 0.26- 0.29 | 0.23 | 0.21- 0.24 | -0.08 | -0.10- -0.06 | -0.08 | -0.10- -0.06 |
| **Variance** |  |  |  |  |  |  |  |  |
| Variance: slope | 0.85 | 0.79- 0.91 | 0.82 | 0.76- 0.88 | 0.37 | 0.34- 0.41 | 0.58 | 0.54- 0.63 |
| Variance: intercept | 1.94 | 1.79- 2.11 | 1.96 | 1.80- 2.13 | 1.53 | 1.41- 1.66 | 1.88 | 1.75- 2.01 |
| Covariance | 0.52 | 0.45- 0.59 | 0.71 | 0.65- 0.78 | 0.54 | 0.49- 0.58 | 0.64 | 0.59- 0.70 |

Supplementary table 3. Unadjusted longitudinal mixed effect model of the association between individual ACEs and BMI/FMI trajectories

|  | BMI trajectories (5- 17 years) | | | | FMI trajectories (7- 17 years) | | | |
| --- | --- | --- | --- | --- | --- | --- | --- | --- |
| Individual ACEs (ref: no) | Boys | | Girls | | Boys | | Girls | |
|  | β | 95% CI | β | 95% CI | β | 95% CI | β | 95% CI |
| Parental separation | 0.09 | -0.20- 0.37 | 0.03 | -0.28- 0.33 | 0.26* | 0.01- 0.51 | 0.03 | -0.25- 0.31 |
| Parental separation*time | 0.14 | -0.04- 0.32 | 0.25* | 0.07- 0.44 | 0.05 | -0.10- 0.20 | 0.20* | 0.03- 0.37 |
| Time/slope | 0.66 | 0.59- 0.72 | 1.10 | 1.04- 1.17 | 0.40 | 0.34- 0.47 | 1.34 | 1.27- 1.40 |
| Time squared | 0.27 | 0.26- 0.29 | 0.23 | 0.21- 0.24 | -0.08 | -0.10- -0.06 | -0.08 | -0.10- -0.06 |
| Intercept | 16.21 | 16.14-16.28 | 15.97 | 15.90-16.04 | 3.45 | 3.39- 3.50 | 3.72 | 3.66- 3.77 |
| Parental depression | 0.19* | 0.07- 0.32 | 0.04 | -0.09- 0.17 | 0.21* | 0.10- 0.32 | 0.11 | -0.01- 0.23 |
| Parental depression*time | 0.15* | 0.07- 0.23 | 0.13* | 0.05- 0.20 | 0.09* | 0.03- 0.15 | 0.09* | 0.02- 0.16 |
| Time/slope | 0.61 | 0.54- 0.68 | 1.07 | 0.99- 1.14 | 0.37 | 0.30- 0.44 | 1.31 | 1.24- 1.38 |
| Time squared | 0.27 | 0.26- 0.29 | 0.23 | 0.21- 0.24 | -0.08 | -0.10- -0.06 | -0.08 | -0.10- -0.06 |
| Intercept | 16.15 | 16.07-16.23 | 15.96 | 15.88-16.04 | 3.39 | 3.32- 3.45 | 3.68 | 3.61- 3.75 |
| Parental drug use | 0.05 | -0.17- 0.26 | -0.10 | -0.32- 0.12 | -0.06 | -0.25- 0.13 | -0.06 | -0.25- 0.14 |
| Parental drug use*time | 0.01 | -0.12- 0.15 | -0.05 | -0.18- 0.08 | 0.02 | -0.08- 0.13 | -0.07 | -0.19- 0.05 |
| Time/slope | 0.66 | 0.59- 0.73 | 1.12 | 1.05- 1.19 | 0.40 | 0.34- 0.47 | 1.35 | 1.28- 1.42 |
| Time squared | 0.27 | 0.26- 0.29 | 0.23 | 0.21- 0.24 | -0.08 | -0.10- -0.06 | -0.08 | -0.10- -0.06 |
| Intercept | 16.21 | 16.14-16.28 | 15.98 | 15.91-16.05 | 3.46 | 3.41- 3.52 | 3.72 | 3.66- 3.78 |
| Parental alcohol misuse | -0.06 | -0.21- 0.08 | -0.08 | -0.22- 0.07 | -0.18* | -0.30- -0.06 | -0.18* | -0.31- -0.05 |
| Parental alcohol misuse*time | -0.12* | -0.20- -0.03 | -0.18* | -0.27- -0.10 | -0.07* | -0.14- -0.01 | -0.16* | -0.24- -0.08 |
| Time/slope | 0.69 | 0.62- 0.76 | 1.15 | 1.08- 1.22 | 0.42 | 0.35- 0.49 | 1.38 | 1.31-1.45 |
| Time squared | 0.27 | 0.26- 0.29 | 0.23 | 0.21- 0.24 | -0.08 | -0.10- -0.06 | -0.08 | -0.10- -0.06 |
| Intercept | 16.23 | 16.16-16.30 | 15.99 | 15.92-16.06 | 3.50 | 3.44- 3.56 | 3.76 | 3.69- 3.82 |
| Interparental use of force | 0.12 | -0.04- 0.29 | 0.19* | 0.02- 0.36 | 0.13 | -0.02- 0.27 | 0.15 | -0.01- 0.30 |
| Interparental use of force*time | 0.07 | -0.03- 0.17 | -0.01 | -0.10- 0.10 | 0.03 | -0.05- 0.11 | -0.05 | -0.14- 0.04 |
| Time/slope | 0.65 | 0.58- 0.72 | 1.11 | 1.04- 1.18 | 0.40 | 0.33- 0.47 | 1.35 | 1.28- 1.42 |
| Time squared | 0.27 | 0.26- 0.29 | 0.23 | 0.21- 0.24 | -0.08 | -0.10- -0.06 | -0.08 | -0.10- -0.06 |
| Intercept | 16.20 | 16.13-16.27 | 15.94 | 15.87-16.01 | 3.44 | 3.38- 3.50 | 3.69 | 3.63- 3.76 |
| Parental discord | 0.09 | -0.12- 0.31 | 0.12 | -0.13- 0.36 | 0.07 | -0.12- 0.26 | 0.01 | -0.21- 0.23 |
| Parental discord*time | 0.07 | -0.06- 0.21 | -0.07 | -0.22- 0.07 | 0.08 | -0.02 -0.19 | -0.04 | -0.18- 0.09 |
| Time/slope | 0.66 | 0.59- 0.72 | 1.12 | 1.05- 1.19 | 0.40 | 0.33- 0.47 | 1.35 | 1.28- 1.41 |
| Time squared | 0.27 | 0.26- 0.29 | 0.23 | 0.21- 0.24 | -0.08 | -0.10- -0.06 | -0.08 | -0.10- -0.06 |
| Intercept | 16.21 | 16.14-16.27 | 15.97 | 15.90-16.03 | 3.45 | 3.40- 3.51 | 3.72 | 3.66- 3.78 |
| Harsh parenting | -0.12 | -0.25- 0.01 | -0.10 | -0.24- 0.04 | -0.12* | -0.23- 0.01 | -0.10 | -0.23- 0.03 |
| Harsh parenting*time | 0.06 | -0.02- 0.14 | -0.01 | -0.09- 0.08 | 0.03 | -0.03- 0.10 | 0.01 | -0.07- 0.08 |
| Time/slope | 0.64 | 0.57- 0.71 | 1.12 | 1.04- 1.19 | 0.39 | 0.32- 0.46 | 1.34 | 1.27- 1.41 |
| Time squared | 0.27 | 0.26-0.29 | 0.23 | 0.21- 0.24 | -0.08 | -0.10- -0.06 | -0.08 | -0.10- -0.06 |
| Intercept | 16.25 | 16.17-16.33 | 16.00 | 15.92-16.07 | 3.50 | 3.43- 3.56 | 3.74 | 3.68- 3.81 |
| Physical punishment | 0.15 | -0.01- 0.31 | -0.07 | -0.26- 0.11 | 0.02 | -0.12- 0.16 | -0.02 | -0.19- 0.15 |
| Physical punishment*time | 0.02 | -0.08- 0.11 | 0.14* | 0.03- 0.26 | 0.03 | -0.05- 0.11 | 0.12* | 0.02- 0.22 |
| Time/slope | 0.66 | 0.59- 0.73 | 1.10 | 1.03- 1.16 | 0.40 | 0.33- 0.47 | 1.33 | 1.26- 1.40 |
| Time squared | 0.27 | 0.26- 0.29 | 0.23 | 0.21- 0.24 | -0.08 | -0.10- -0.06 | -0.08 | -0.10- -0.06 |
| Intercept | 16.19 | 16.12-16.26 | 15.98 | 15.91-16.05 | 3.46 | 3.40- 3.52 | 3.72 | 3.66- 3.78 |

Supplementary table 4. Adjusted longitudinal mixed effect model of the interaction effect between ACE score and poverty on BMI/ FMI trajectories

|  | BMI trajectories (5- 17 years) | | | | FMI trajectories (7- 17 years) | | | |
| --- | --- | --- | --- | --- | --- | --- | --- | --- |
|  | Boys | | Girls | | Boys | | Girls | |
|  | β | 95% CI | β | 95% CI | β | 95% CI | β | 95% CI |
| **Baseline** |  |  |  |  |  |  |  |  |
| 0 ACEs (ref) |  |  |  |  |  |  |  |  |
| 1 ACE | -0.04 | -0.21- 0.13 | 0.02 | -0.14- 0.19 | -0.01 | -0.15- 0.14 | -0.01 | -0.16- 0.13 |
| 2 ACEs | 0.04 | -0.14- 0.22 | -0.04 | -0.22- 0.14 | 0.03 | -0.12- 0.19 | -0.06 | -0.23- 0.10 |
| 3+ ACEs | 0.14 | -0.07- 0.34 | -0.05 | -0.26- 0.17 | 0.03 | -0.15- 0.20 | -0.03 | -0.22- 0.16 |
| Poverty: No (ref) |  |  |  |  |  |  |  |  |
| Poverty: Yes | -0.18 | -0.52- 0.16 | -0.03 | -0.36- 0.31 | -0.30* | -0.59- -0.01 | 0.01 | -0.30- 0.31 |
| Intercept (5/7 years) | 16.51 | 16.12-16.89 | 16.30 | 15.92-16.68 | 4.01 | 3.66- 4.35 | 3.93 | 3.58- 4.29 |
| **Rate of change** |  |  |  |  |  |  |  |  |
| 0 ACEs (ref) |  |  |  |  |  |  |  |  |
| 1 ACE | 0.08 | -0.02- 0.19 | 0.05 | -0.05- 0.15 | 0.06 | -0.02- 0.15 | 0.06 | -0.03- 0.15 |
| 2 ACEs | 0.07 | -0.04- 0.18 | 0.01 | -0.11- 0.11 | 0.03 | -0.06- 0.12 | 0.03 | -0.07- 0.13 |
| 3+ ACEs | 0.13* | 0.01-0.26 | 0.03 | -0.10- 0.17 | 0.10* | 0.01- 0.20 | -0.01 | -0.13- 0.11 |
| Poverty: No (ref) |  |  |  |  |  |  |  |  |
| Poverty: Yes | 0.18 | -0.03- 0.39 | 0.14 | -0.07- 0.34 | 0.22* | 0.06- 0.39 | 0.11 | -0.08- 0.29 |
| Time/Slope | 0.57 | 0.47- 0.67 | 1.05 | 0.96- 1.15 | 0.33 | 0.25- 0.42 | 1.29 | 1.20- 1.38 |
| Time squared | 0.27 | 0.26- 0.29 | 0.23 | 0.21- 0.24 | -0.08 | -0.10- -0.06 | -0.08 | -0.10- -0.06 |
| **Interaction** |  |  |  |  |  |  |  |  |
| Baseline |  |  |  |  |  |  |  |  |
| 0 ACEs*poverty (ref) |  |  |  |  |  |  |  |  |
| 1 ACE*poverty | -0.16 | -0.60- 0.28 | -0.04 | -0.47- 0.39 | 0.01 | -0.37- 0.38 | 0.18 | -0.22- 0.57 |
| 2 ACEs*poverty | 0.11 | -0.35- 0.57 | -0.28 | -0.74- 0.18 | 0.28 | -0.12- 0.68 | -0.12 | -0.54- 0.30 |
| 3+ ACEs*poverty | 0.02 | -0.46- 0.50 | 0.38 | -0.12- 0.88 | 0.32 | -0.10- 0.74 | 0.33 | -0.12- 0.78 |
| Rate of change |  |  |  |  |  |  |  |  |
| 0 ACEs*poverty (ref) |  |  |  |  |  |  |  |  |
| 1 ACE*poverty | -0.05 | -0.33- 0.22 | 0.17 | -0.10- 0.44 | -0.06 | -0.28- 0.17 | 0.11 | -0.13- 0.36 |
| 2 ACEs*poverty | -0.07 | -0.35- 0.22 | 0.07 | -0.22- 0.36 | -0.17 | -0.40- 0.07 | 0.06 | -0.20- 0.32 |
| 3+ ACEs*poverty | -0.04 | -0.34- 0.26 | 0.07 | -0.23- 0.38 | -0.09 | -0.33- 0.15 | 0.06 | -0.22- 0.35 |
| **Variance** |  |  |  |  |  |  |  |  |
| Variance: slope | 0.84 | 0.79- 0.90 | 0.81 | 0.76- 0.87 | 0.37 | 0.33- 0.40 | 0.58 | 0.54- 0.63 |
| Variance: intercept | 1.73 | 1.59- 1.89 | 1.69 | 1.55- 1.85 | 1.34 | 1.23- 1.46 | 1.66 | 1.54- 1.78 |
| Covariance | 0.42 | 0.35- 0.49 | 0.60 | 0.53- 0.67 | 0.46 | 0.42- 0.51 | 0.54 | 0.48- 0.59 |

Supplementary table 5. Adjusted longitudinal mixed effect model of the interaction effect of individual ACEs and poverty on BMI/ FMI trajectories

|  | BMI trajectories (5- 17 years) | | | | FMI trajectories (7- 17 years) | | | |
| --- | --- | --- | --- | --- | --- | --- | --- | --- |
| Individual ACEs/poverty (ref: no) | Boys | | Girls | | Boys | | Girls | |
|  | β | 95% CI | β | 95% CI | β | 95% CI | β | 95% CI |
| Parental separation | -0.25 | -0.69- 0.18 | 0.12 | -0.33- 0.58 | 0.13 | -0.25- 0.51 | -0.11 | -0.52- 0.31 |
| Poverty | -0.29* | -0.48- -0.10 | -0.01 | -0.20- 0.18 | -0.23* | -0.40- -0.07 | 0.09 | -0.08- 0.26 |
| Parental separation*time | 0.10 | -0.17- 0.38 | 0.18 | -0.10- 0.47 | -0.03 | -0.26- 0.19 | 0.20 | -0.06- 0.47 |
| Poverty*time | 0.13* | 0.02- 0.24 | 0.20* | 0.10- 0.31 | 0.15* | 0.06- 0.23 | 0.16* | 0.06- 0.26 |
| Parental separation*poverty | 0.76* | 0.18- 1.34 | -0.26 | -0.87- 0.35 | 0.34 | -0.16- 0.85 | 0.10 | -0.45- 0.66 |
| Parental separation*poverty*time | -0.03 | -0.40- 0.34 | -0.04 | -0.43- 0.34 | 0.03 | -0.29- 0.33 | -0.13 | -0.48- 0.23 |
| Time/slope | 0.64 | 0.59- 0.71 | 1.07 | 1.00- 1.14 | 0.38 | 0.31- 0.45 | 1.31 | 1.25- 1.38 |
| Time squared | 0.27 | 0.26- 0.29 | 0.23 | 0.21- 0.24 | -0.08 | -0.10- -0.06 | -0.08 | -0.10- -0.06 |
| Intercept | 16.51 | 16.14-16.88 | 16.31 | 15.94-16.68 | 4.00 | 3.66- 4.33 | 3.94 | 3.60- 4.28 |
| Parental depression | 0.16* | 0.03- 0.30 | -0.09 | -0.23- 0.04 | 0.15* | 0.04- 0.27 | -0.01 | -0.14- 0.11 |
| Poverty | -0.12 | -0.34- 0.10 | -0.20 | -0.43- 0.03 | -0.17 | -0.36- 0.02 | -0.01 | -0.22- 0.20 |
| Parental depression*time | 0.14* | 0.05- 0.22 | 0.12* | 0.03- 0.20 | 0.08* | 0.02- 0.15 | 0.08* | 0.01- 0.16 |
| Poverty*time | 0.11 | -0.02- 0.24 | 0.22* | 0.09- 0.36 | 0.15* | 0.04- 0.25 | 0.16* | 0.04- 0.29 |
| Parental depression *poverty | -0.19 | -0.51- 0.14 | 0.37* | 0.04- 0.70 | 0.00 | -0.28- 0.29 | 0.23 | -0.07- 0.52 |
| Parental depression *poverty*time | 0.03 | -0.17- 0.24 | -0.04 | -0.24- 0.16 | -0.02 | -0.19- 0.14 | -0.02 | -0.20- 0.17 |
| Time/slope | 0.59 | 0.52- 0.67 | 1.04 | 0.96- 1.11 | 0.35 | 0.28- 0.42 | 1.29 | 1.22- 1.36 |
| Time squared | 0.27 | 0.26- 0.29 | 0.23 | 0.21- 0.24 | -0.08 | -0.10- -0.06 | -0.08 | -0.10- -0.06 |
| Intercept | 16.47 | 16.10-16.84 | 16.33 | 15.96-16.71 | 3.97 | 3.63- 4.30 | 3.92 | 3.57- 4.26 |
| Parental drug use | 0.15 | -0.09- 0.39 | -0.03 | -0.27- 0.21 | 0.02 | -0.19- 0.23 | -0.01 | -0.22- 0.21 |
| Poverty | -0.18 | -0.36- 0.01 | -0.05 | -0.23- 0.14 | -0.15 | -0.32- 0.01 | 0.08 | -0.09- 0.25 |
| Parental drug use*time | 0.01 | -0.14- 0.16 | -0.09 | -0.24- 0.06 | 0.02 | -0.10- 0.14 | -0.10 | -0.24- 0.03 |
| Poverty*time | 0.14* | 0.04- 0.25 | 0.21* | 0.10- 0.31 | 0.15* | 0.06- 0.24 | 0.16* | 0.06- 0.25 |
| Parental drug use*poverty | -0.15 | -0.65- 0.34 | 0.10 | -0.41- 0.62 | -0.05 | -0.48- 0.39 | 0.09 | -0.37- 0.56 |
| Parental drug use*poverty*time | -0.04 | -0.35- 0.27 | 0.15 | -0.17- 0.47 | -0.04 | -0.29- 0.21 | 0.11 | -0.18- 0.41 |
| Time/slope | 0.64 | 0.57- 0.71 | 1.08 | 1.01- 1.15 | 0.39 | 0.31- 0.45 | 1.33 | 1.26- 1.39 |
| Time squared | 0.27 | 0.26- 0.29 | 0.23 | 0.21- 0.24 | -0.08 | -0.10- -0.06 | -0.08 | -0.10- -0.06 |
| Intercept | 16.52 | 16.15-16.89 | 16.32 | 15.95-16.69 | 4.04 | 3.71- 4.37 | 3.93 | 3.59- 4.27 |
| Parental alcohol misuse | 0.02 | -0.13- 0.17 | -0.08 | -0.23- 0.08 | -0.06 | -0.19- 0.07 | -0.12 | -0.25- 0.02 |
| Poverty | -0.18 | -0.37- 0.01 | -0.10 | -0.29- 0.09 | -0.16 | -0.33- 0.01 | 0.07 | -0.11- 0.24 |
| Parental alcohol misuse*time | -0.08 | -0.18- 0.01 | -0.17* | -0.26- -0.08 | -0.06* | -0.13- -0.02 | -0.15* | -0.23- -0.07 |
| Poverty*time | 0.16* | 0.05- 0.27 | 0.21* | 0.10- 0.32 | 0.15* | 0.06- 0.24 | 0.15* | 0.05- 0.25 |
| Parental alcohol misuse*poverty | -0.06 | -0.48- 0.37 | 0.38 | -0.06- 0.82 | -0.01 | -0.37- 0.37 | 0.17 | -0.24- 0.57 |
| Parental alcohol misuse*poverty*time | -0.17 | -0.45- 0.10 | 0.00 | -0.27- 0.27 | -0.06 | -0.28- 0.16 | 0.03 | -0.22- 0.27 |
| Time/slope | 0.66 | 0.59- 0.73 | 1.12 | 1.04- 1.19 | 0.39 | 0.32- 0.47 | 1.35 | 1.28-1.42 |
| Time squared | 0.27 | 0.26- 0.29 | 0.23 | 0.21- 0.24 | -0.08 | -0.10- -0.06 | -0.08 | -0.10- -0.06 |
| Intercept | 16.54 | 16.17-16.91 | 16.33 | 15.96-16.70 | 4.05 | 3.72- 4.38 | 3.95 | 3.61- 4.29 |
| Interparental use of force | 0.17 | -0.01- 0.35 | 0.20* | 0.02- 0.38 | 0.14 | -0.02- 0.29 | 0.15 | -0.01- 0.31 |
| Poverty | -0.17 | -0.36- 0.02 | -0.06 | -0.25- 0.13 | -0.17* | -0.34- -0.01 | 0.06 | -0.11- 0.23 |
| Interparental use of force*time | 0.08 | -0.03- 0.19 | -0.03 | -0.14- 0.08 | 0.04 | -0.05- 0.12 | -0.06 | -0.16- 0.04 |
| Poverty*time | 0.15* | 0.04- 0.26 | 0.20* | 0.09- 0.31 | 0.16* | 0.07- 0.25 | 0.16* | 0.06- 0.26 |
| Interparental use of force*poverty | -0.14 | -0.56- 0.29 | 0.11 | -0.32- 0.54 | 0.07 | -0.30- 0.45 | 0.18 | -0.20- 0.56 |
| Interparental use of force*poverty*time | -0.05 | -0.32- 0.22 | 0.10 | -0.17- 0.37 | -0.06 | -0.28- 0.16 | 0.06 | -0.19- 0.30 |
| Time/slope | 0.63 | 0.56- 0.70 | 1.09 | 1.01- 1.15 | 0.38 | 0.31- 0.44 | 1.33 | 1.26- 1.40 |
| Time squared | 0.27 | 0.26- 0.29 | 0.23 | 0.21- 0.24 | -0.08 | -0.10- -0.06 | -0.08 | -0.10- -0.06 |
| Intercept | 16.51 | 16.15-16.88 | 16.29 | 15.92-16.66 | 4.02 | 3.69- 4.35 | 3.91 | 3.57- 4.25 |
| Parental discord | 0.19 | -0.05- 0.43 | 0.02 | -0.24- 0.29 | 0.11 | -0.09- 0.31 | -0.11 | -035- 0.12 |
| Poverty | -0.14 | -0.33- 0.04 | -0.06 | -0.24- 0.12 | -0.14 | -0.30- 0.02 | 0.07 | -0.10- 0.24 |
| Parental discord*time | 0.03 | -0.12- 0.18 | -0.05 | -0.22- 0.11 | 0.05 | -0.07- 0.17 | -0.02 | -0.17- 0.13 |
| Poverty*time | 0.12* | 0.02- 0.23 | 0.24* | 0.13- 0.34 | 0.13* | 0.04- 0.22 | 0.18* | 0.09- 0.28 |
| Parental discord*poverty | -0.50 | -1.03- 0.03 | 0.29 | -0.29- 0.88 | -0.22 | -0.68- 0.25 | 0.32 | -0.20- 0.84 |
| Parental discord*poverty*time | 0.15 | -0.19- 0.48 | -0.16 | -0.52- 0.20 | 0.13 | -0.13- 0.40 | -0.16 | -0.49- 0.17 |
| Time/slope | 0.64 | 0.57- 0.71 | 1.08 | 1.01- 1.15 | 0.38 | 0.31- 0.45 | 1.32 | 1.25- 1.39 |
| Time squared | 0.27 | 0.26- 0.29 | 0.23 | 0.21- 0.24 | -0.08 | -0.10- -0.06 | -0.08 | -0.10- -0.06 |
| Intercept | 16.52 | 16.15-16.89 | 16.31 | 15.94-16.67 | 4.03 | 3.70- 4.36 | 3.94 | 3.60- 4.27 |
| Harsh parenting | -0.15* | -0.28- -0.01 | -0.07 | -0.22- 0.07 | -0.11 | -0.23- 0.01 | -0.05 | -0.19- 0.08 |
| Poverty | -0.26* | -0.46- -0.06 | -0.01 | -0.20- 0.19 | -0.22* | -0.40- -0.05 | 0.12 | -0.06- 0.30 |
| Harsh parenting*time | 0.08 | -0.01- 0.16 | -0.01 | -0.10- 0.08 | 0.05 | -0.02- 0.12 | -0.01 | -0.08- 0.08 |
| Poverty*time | 0.16* | 0.04- 0.28 | 0.20* | 0.09- 0.32 | 0.17* | 0.07- 0.26 | 0.14 | 0.03- 0.24 |
| Harsh parenting*poverty | 0.23 | -0.13- 0.60 | -0.15 | -0.54- 0.24 | 0.24 | -0.08- 0.55 | -0.11 | -0.46- 0.24 |
| Harsh parenting*poverty*time | -0.05 | -0.28- 0.18 | 0.08 | -0.16- 0.32 | -0.07 | -0.26- 0.12 | 0.13 | -0.09- 0.34 |
| Time/slope | 0.62 | 0.54- 0.69 | 1.08 | 1.01- 1.15 | 0.36 | 0.29- 0.44 | 1.32 | 1.25- 1.39 |
| Time squared | 0.27 | 0.26-0.29 | 0.23 | 0.21- 0.24 | -0.08 | -0.10- -0.06 | -0.08 | -0.10- -0.06 |
| Intercept | 16.59 | 16.22-16.96 | 16.32 | 15.95-16.69 | 4.07 | 3.74- 4.40 | 3.93 | 3.59- 4.27 |
| Physical punishment | 0.07 | -0.10- 0.23 | -0.01 | -0.21- 0.19 | -0.06 | -0.20- 0.09 | 0.02 | -0.15- 0.20 |
| Poverty | -0.23* | -0.42- -0.04 | -0.01 | -0.20- 0.17 | -0.19* | -0.36- -0.03 | 0.12 | -0.05- 0.29 |
| Physical punishment*time | 0.04 | -0.07- 0.14 | 0.16* | 0.03- 0.28 | 0.05 | -0.04- 0.13 | 0.14* | 0.03- 0.25 |
| Poverty*time | 0.16* | 0.05- 0.27 | 0.23* | 0.12- 0.34 | 0.17 | 0.08- 0.26 | 0.18* | 0.08- 0.28 |
| Physical punishment*poverty | 0.22 | -0.18- 0.63 | -0.17 | -0.66- 0.32 | 0.19 | -0.17- 0.54 | -0.18 | -0.62- 0.26 |
| Physical punishment*poverty*time | -0.11 | -0.37- 0.14 | -0.09 | -0.39- 0.21 | -0.12 | -0.33- 0.09 | -0.12 | -0.39- 0.15 |
| Time/slope | 0.63 | 0.56- 0.71 | 1.06 | 0.99- 1.13 | 0.37 | 0.30- 0.44 | 1.30 | 1.23- 1.37 |
| Time squared | 0.27 | 0.26- 0.29 | 0.23 | 0.21- 0.24 | -0.08 | -0.10- -0.06 | -0.08 | -0.10- -0.06 |
| Intercept | 16.51 | 16.15-16.88 | 16.31 | 15.94-16.68 | 4.04 | 3.72- 4.38 | 3.92 | 3.58- 4.26 |


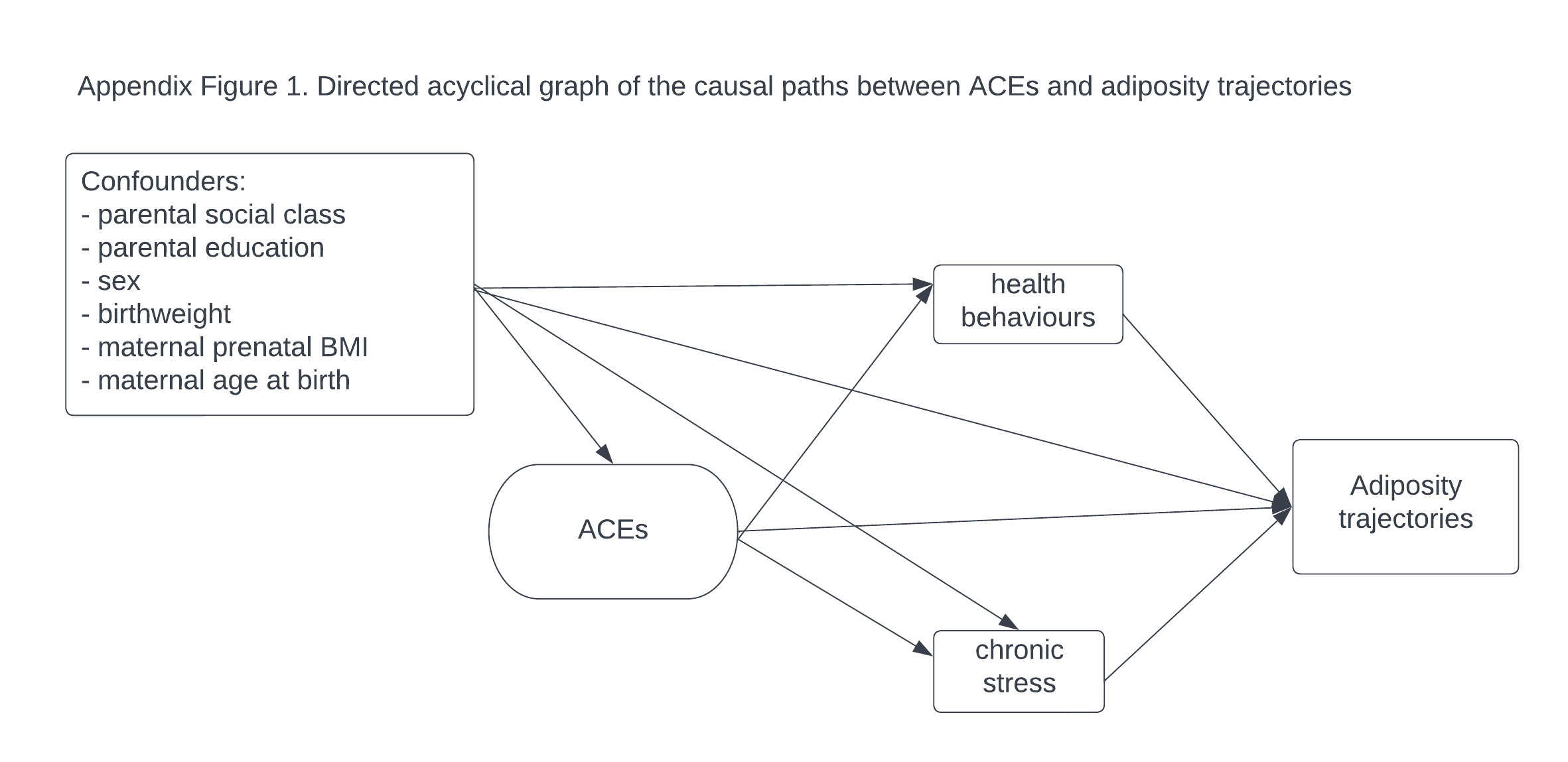

Supplement: Supplementary file 1 — Supplemental materials [file 41366_2022_1185_MOESM1_ESM.docx]
