# Supplementary material for: Adverse childhood experiences, child poverty, and adiposity trajectories from childhood to adolescence: evidence from the Millennium Cohort Study
Source: Int J Obes (Lond). 2022 Jul 15;46(10):1792–800. doi: 10.1038/s41366-022-01185-1 (PMC9492536; doi:10.1038/s41366-022-01185-1)
Supplement: Supplementary file 2 — STROBE checklist [file 41366_2022_1185_MOESM2_ESM.doc]

STROBE Statement—Checklist of items that should be included in reports of ***cohort studies***

|  | Item No | Recommendation |
| --- | --- | --- |
| **Title and abstract** | 1 | (*a*) Indicate the study’s design with a commonly used term in the title or the abstract  *Cohort study as stated in the title (p.1) and abstract (p.2).* |
|  |  | (*b*) Provide in the abstract an informative and balanced summary of what was done and what was found  *Provided in the abstract (p.2-3).* |
| Introduction | | |
| Background/rationale | 2 | Explain the scientific background and rationale for the investigation being reported  *Included in the introduction section (p.4-6).* |
| Objectives | 3 | State specific objectives, including any prespecified hypotheses  *Included in the introduction section (p.6).* |
| Methods | | |
| Study design | 4 | Present key elements of study design early in the paper  *Included in the methods section (p.7).* |
| Setting | 5 | Describe the setting, locations, and relevant dates, including periods of recruitment, exposure, follow-up, and data collection  *Included in the methods section (p.7-10).* |
| Participants | 6 | (*a*) Give the eligibility criteria, and the sources and methods of selection of participants. Describe methods of follow-up  *Included in the methods section (p.7, 10-11).* |
|  |  | (*b*) For matched studies, give matching criteria and number of exposed and unexposed  *Not applicable.* |
| Variables | 7 | Clearly define all outcomes, exposures, predictors, potential confounders, and effect modifiers. Give diagnostic criteria, if applicable  *Included in the methods section (p.7-10).* |
| Data sources/ measurement | 8* | For each variable of interest, give sources of data and details of methods of assessment (measurement). Describe comparability of assessment methods if there is more than one group  *Included in the methods section (p.7-10).* |
| Bias | 9 | Describe any efforts to address potential sources of bias  *Addressed in the limitations paragraph in the discussion section (p.18-19).* |
| Study size | 10 | Explain how the study size was arrived at  *Included in the methods section (p.10-11) and illustrated in Figure.1.* |
| Quantitative variables | 11 | Explain how quantitative variables were handled in the analyses. If applicable, describe which groupings were chosen and why  *Included in the methods section (p.7-10).* |
| Statistical methods | 12 | (*a*) Describe all statistical methods, including those used to control for confounding  *Included in the methods section (p.10-11).* |
|  |  | (*b*) Describe any methods used to examine subgroups and interactions  *Included in the methods section (p.10-11).* |
|  |  | (*c*) Explain how missing data were addressed  *Included in the methods section (p.10-11).* |
|  |  | (*d*) If applicable, explain how loss to follow-up was addressed  *Complete case analysis was used, and this is discussed in the limitations paragraph (p.18-19).* |
|  |  | (*e*) Describe any sensitivity analyses  *Not applicable.* |
| Results | | |
| Participants | 13* | (a) Report numbers of individuals at each stage of study—eg numbers potentially eligible, examined for eligibility, confirmed eligible, included in the study, completing follow-up, and analysed  *Shown in a flow diagram (Figure.1).* |
|  |  | (b) Give reasons for non-participation at each stage  *Not applicable.* |
|  |  | (c) Consider use of a flow diagram  *Figure 1 is a flow diagram showing the derivation of the sample.* |
| Descriptive data | 14* | (a) Give characteristics of study participants (eg demographic, clinical, social) and information on exposures and potential confounders  *Included in the results section (p.12).* |
|  |  | (b) Indicate number of participants with missing data for each variable of interest  *Included in Table 1.* |
|  |  | (c) Summarise follow-up time (eg, average and total amount)  *Included in the methods section (p.7).* |
| Outcome data | 15* | Report numbers of outcome events or summary measures over time  *Included in the results section (p.12) and summarised in Table 1.* |
| Main results | 16 | (*a*) Give unadjusted estimates and, if applicable, confounder-adjusted estimates and their precision (eg, 95% confidence interval). Make clear which confounders were adjusted for and why they were included  *Included in the results section (p.12-13,) and summarised in Table 2-3. The confounders included was stated in the methods section (p.10). Unadjusted estimates were presented in the supplementary tables due to limitation of space.* |
|  |  | (*b*) Report category boundaries when continuous variables were categorized  *Not applicable.* |
|  |  | (*c*) If relevant, consider translating estimates of relative risk into absolute risk for a meaningful time period  *Not applicable.* |
| Other analyses | 17 | Report other analyses done—eg analyses of subgroups and interactions, and sensitivity analyses  *The analysis was stratified by sex and the results were reported in the results section (p.12-13) and Table 2-3. The results of interactions between ACEs and poverty were included in the results section (p.14) and the supplementary table 2 and 3.* |
| Discussion | | |
| Key results | 18 | Summarise key results with reference to study objectives  *Included in the discussion section (p.15).* |
| Limitations | 19 | Discuss limitations of the study, taking into account sources of potential bias or imprecision. Discuss both direction and magnitude of any potential bias  *Included a limitations paragraph in the discussion section (p.18-19).* |
| Interpretation | 20 | Give a cautious overall interpretation of results considering objectives, limitations, multiplicity of analyses, results from similar studies, and other relevant evidence  *Included in the discussion section (p.15-19).* |
| Generalisability | 21 | Discuss the generalisability (external validity) of the study results  *Included in the discussion section (p.18).* |
| Other information | | |
| Funding | 22 | Give the source of funding and the role of the funders for the present study and, if applicable, for the original study on which the present article is based  *Stated in acknowledgment (p.21).* |

*Give information separately for exposed and unexposed groups.

**Note:** An Explanation and Elaboration article discusses each checklist item and gives methodological background and published examples of transparent reporting. The STROBE checklist is best used in conjunction with this article (freely available on the Web sites of PLoS Medicine at http://www.plosmedicine.org/, Annals of Internal Medicine at http://www.annals.org/, and Epidemiology at http://www.epidem.com/). Information on the STROBE Initiative is available at http://www.strobe-statement.org.
